# The threshold effect of triglyceride glucose index on diabetic kidney disease risk in patients with type 2 diabetes: unveiling a non-linear association

**DOI:** 10.3389/fendo.2024.1411486

**Published:** 2024-06-13

**Authors:** Huabin Wang, Guangming Chen, Dongmei Sun, Yongjun Ma

**Affiliations:** ^1^ Department of Clinical Laboratory, Affiliated Jinhua Hospital, Zhejiang University School of Medicine, Jinhua, Zhejiang, China; ^2^ Department of General Practice, Affiliated Jinhua Hospital, Zhejiang University School of Medicine, Jinhua, China

**Keywords:** diabetic kidney disease, insulin resistance, triglyceride glucose index, threshold effect, type 2 diabetes

## Abstract

**Background:**

Previous studies have confirmed that the triglyceride glucose (TyG) index, recognized as a reliable marker of insulin resistance, is an important risk factor for diabetic kidney disease (DKD). However, it is still unclear whether the DKD risk continues to increase linearly with the elevation of TyG index. This study aimed to thoroughly investigated the intrinsic relationship between TyG index and DKD risk in type 2 diabetes (T2D).

**Methods:**

This cross-sectional study included 933 patients with T2D in China, who were categorized into DKD and non-DKD groups and stratified by TyG index levels. Logistic regression analysis identified the independent risk factors for DKD. The association between DKD risk and TyG index was evaluated using the restricted cubic spline (RCS) curves analysis. The R package ‘CatPredi’ was utilized to determine the optimal cut-off point for the relationship between DKD risk and TyG index, followed by threshold effect analysis.

**Results:**

The prevalence of DKD was 33.01%. After adjusting for confounding factors, TyG index was identified as a prominent clinical risk factor for DKD, showing the highest odds ratio (OR 1.57 (1.26 - 1.94), P<0.001). RCS analysis revealed a non-linear relationship with a threshold interval effect between the TyG index and DKD risk. When TyG index ≤ 9.35, DKD risk plateaued at a low level; however, when TyG index > 9.35, DKD risk increased gradually with rising TyG index. Among patients with TyG index > 9.35, each 1-unit increase was associated with a 1.94-fold increased DKD risk (OR=1.94 (1.10 - 3.43), P=0.022).

**Conclusion:**

The DKD risk presented a threshold effect with the increase of TyG index, initially stable at a low level, and then gradually rising when the TyG index is above 9.35.

## 1 Introduction

Diabetic kidney disease (DKD) is now prevalent as a major complication of diabetes and the primary cause of end-stage renal disease (1–3). Presently, the therapies for managing DKD involve the regulation of blood pressure and glucose levels, along with the use of angiotensin-converting enzyme inhibitors (ACEI) and angiotensin-receptor blockers (ARB); nevertheless, the effectiveness of these treatment modalities in halting the advancement of DKD is restricted (4, 5), underscoring a substantial ongoing challenge in preventing and managing the progression of DKD.

The development and progression of DKD are influenced by multiple factors. Genetic variations and prolonged hyperglycemic states are known to activate cellular pathways that exacerbate renal damage (6). Concurrently, chronic inflammation can further amplify this damage, ultimately setting the stage for significant renal impairment (6, 7). Moreover, insulin resistance is also considered to be associated with the clinical symptoms of DKD and may be one of the underlying causes of the histological features of DKD (8). An increasing number of studies have shown that insulin resistance plays an important role in the development and progression of DKD (9–11). Insulin resistance can be identified in the early stages of chronic renal disease, with its intensity escalating as renal function deteriorates (12, 13).

Insulin resistance is commonly associated with elevated levels of triglycerides and fasting glucose in the blood. When insulin resistance occurs, insulin’s normal physiological actions are hindered, leading to increased blood glucose levels. Concurrently, insulin resistance affects the function of adipose tissue, enhancing lipolysis and thus raising triglyceride levels in the bloodstream. Therefore, the triglyceride glucose (TyG) index, calculated as the logarithm of the product of fasting triglycerides and glucose, seeks to provide a simple and effective quantitative measure to reflect an individual’s level of insulin resistance. By integrating these two indicators, the TyG index offers a practical tool for assessing insulin resistance (14, 15). Although the hyperinsulinemic euglycemic glucose clamp test is the gold standard method for evaluating insulin resistance, this technique is expensive and complex to conduct in a clinical setting (16). The TyG index is a novel marker, demonstrating superior performance compared to homeostasis model assessment and aligning well with the high insulin-glucose clamp test (17, 18).

Previous studies have reported that the TyG index is independently associated with DKD (12, 19); however, these studies simply indicated a general trend of a higher risk of DKD with elevated TyG index values, but they did not offer insight into the dynamic relationship between them or confirm whether there was a linear correlation between DKD risk and increasing TyG index values. Therefore, this study aimed to investigate the intrinsic relationship between the TyG index and the risk of DKD in patients with type 2 diabetes (T2D).

## 2 Materials and methods

### 2.1 Study population

This cross-sectional study included 933 patients with T2D who had visited at the Department of Endocrinology, Affiliated Jinhua Hospital, Zhejiang University School of Medicine from September 2020 to July 2021. The inclusion criteria encompassed individuals aged over 18 years who had a diagnosis of T2D. Participants with a previous history of renal diseases other than DKD, severe congenital heart disease, severe heart failure, systemic immune diseases, severe liver diseases, malignant tumors, etc. were excluded. We also excluded participants who were pregnant at the time of data collection, suffered from acute or chronic infections, and/or had important laboratory data missing. The average age of the 933 subjects in this study was 59.97 ± 12.85 years, with a median diabetes duration of 8 (2, 13) years. Female subjects accounted for 41.16%, hypertensive patients accounted for 55.09%, 24.22% of subjects used ACEI/ARB medication, 35.69% of subjects had insulin therapy, and 25.40% of subjects had lipid lowering agents usage. The present study followed the tenets of the Declaration of Helsinki and was approved by the Ethics Committee of Affiliated Jinhua Hospital, Zhejiang University School of Medicine (ethical approval number: (Res) 2021-Ethical Review-75–01), on July 21, 2021. According to the regulations of the Ethics Committee, the consent for participation is not necessary for this retrospective cross-sectional study.

### 2.2 Clinical and laboratory parameters

The general clinical information of the participants such as gender, age, hypertension, the usage of ACEI/ARB medication, and the diabetic duration, height, weight, diastolic blood pressure (DBP), systolic blood pressure (SBP), the usage of insulin and the lipid lowering agents were collected through the electronic medical record system. The fasting blood samples and the first morning urine samples were collected the morning of the day after admission and then analyzed in the department of clinical laboratory. Glycated hemoglobin (HbA1c) levels were assessed using the BIO-RAD D-100 analyzer. Low-density lipoprotein cholesterol (LDL-C), high-density lipoprotein cholesterol (HDL-C), triglycerides (TG), fasting blood glucose (FBG), serum creatinine, urine creatinine and urine albumin were measured using the Beckman Coulter automatic biochemical analyzer (AU5800) and its original reagents.

We subsequently computed the urine albumin-to-creatinine ratio (ACR), eGFR, TyG index, body mass index (BMI), and TyG-BMI index. The ACR was defined as urine albumin/urine creatinine. The Xiangya equation based on serum creatinine was used to calculate eGFR values (20). The TyG index was calculated using the formula Ln [TG (mg/dL) × FBG (mg/dL)/2] (21). BMI was defined as the weight/square of the height. The TyG-BMI index was determined by multiplying the TyG index by BMI (19). In this study, DKD was defined as eGFR below 60 ml/min/1.73m^2^ and/or ACR exceeding 30 mg/g (5).

### 2.3 Statistical analysis

In the present study, SPSS 26.0 statistical software, R software (3.6.3 version) and the Deepwise and Beckman Coulter DxAI platform (https://dxonline.deepwise.com/) were used to analyze the data. The continuous variables with a normal distribution were presented as mean ± standard deviation, whereas the continuous variables with a skewed distribution were presented as median with interquartile range (Q1 - Q3). Categorical variables were reported as frequency and percentage (%). The participants were categorized into DKD and non-DKD groups and stratified by TyG index levels (in tertiles). Between-group comparisons were performed using t-tests, one-way analysis of variance (ANOVA), chi-square tests, and Mann-Whitney U tests, as appropriate. Independent clinical risk factors of DKD were identified using multivariate logistic regression analysis. The forest plot was performed to demonstrate the influence of clinical risk factors on DKD risk. The association between various TyG index levels (in tertiles) and the risk of DKD was assessed by conducting univariate and multivariate logistic analyses. The restricted cubic spline (RCS) curve analysis revealed a non-linear relationship between the TyG index and the risk of DKD. The optimal cut-off point for the nonlinear relationship between the TyG index and DKD risk was determined using the R package ‘CatPredi’. A threshold effect analysis of TyG index on the risk of DKD was performed using logistic regression. Statistical significance was defined as P < 0.05.

## 3 Results

### 3.1 Clinical characteristics of the subjects categorized by the TyG index

The study population was divided into tertiles based on TyG index levels: the first group (tertile 1) comprised 311 subjects with a TyG index < 8.70, the second group (tertile 2) consisted of 311 participants with a TyG index between 8.70 and 9.28, and the third group (tertile 3) included 311 patients with a TyG index > 9.28. The main demographic and clinical characteristics of these groups were presented in **Table 1**. Compared to those in the lowest tertile, the subjects in the higher tertiles of the TyG index were younger; had shorter diabetic duration; had lower levels of HDL-C; had higher DBP, BMI, HbA1c, LDL-C, TG, FBG, TyG-BMI, and ACR (all P for trend < 0.05). Importantly, despite the lack of statistical differences among the three groups, individuals in the higher tertiles exhibited a higher prevalence of DKD in comparison to those in the lowest tertile of the TyG index.

**Table 1:** Demographic and clinical characteristics of participants categorized by the TyG index.

Parameters	Tertile 1 (n=311)	Tertile 2 (n=311)	Tertile 3 (n=311)	*P* value
Age (year)	63.38 ± 12.66	60.52 ± 11.48	56.01 ± 13.24	<0.001
Female, n (%)	132 (42.44)	129 (41.48)	123 (39.55)	0.757
Hypertension, n (%)	176 (56.59)	176 (56.59)	16 2(52.09)	0.428
ACEI/ARB use, n (%)	74 (23.79)	75 (24.12)	77 (24.76)	0.96
Diabetic duration (year)	10 (3, 15)	8 (3, 14)	7 (1, 12)	0.004
SBP (mmHg)	137.67 ± 19.31	137.958(19.713)	139.048(18.253)	0.639
DBP (mmHg)	75.27 ± 10.91	79.24 ± 12.38	81.54 ± 11.64	<0.001
BMI	23.96 ± 3.16	24.82 ± 3.13	25.87 ± 4.64	<0.001
HbA1c (%)	7.41 ± 1.74	8.11 ± 2.13	9.11 ± 2.20	<0.001
LDL-C (mmol/l)	2.50 ± 0.81	2.86 ± 0.79	3.33 ± 0.92	<0.001
HDL-C (mmol/l)	1.25 ± 0.34	1.12 ± 0.24	1.14 ± 0.42	<0.001
TG (mmol/l)	0.94 ± 0.27	1.45 ± 0.38	2.97 ± 2.19	<0.001
FBG (mmol/l)	5.85 ± 1.49	7.47 ± 2.15	9.81 ± 2.99	<0.001
TyG-BMI	197.29 ± 34.00	222.48 ± 31.08	255.45 ± 49.04	<0.001
TyG index	8.31 ± 0.31	8.99 ± 0.17	9.86 ± 0.52	<0.001
Serum creatinine (μmol/L)	72 (63, 87)	72 (64, 81)	73 (62, 85)	0.552
eGFR (ml/min/1.73 m2)	74.81 ± 2.12	77.10 ± 11.08	77.98 ± 14.48	0.006
ACR (mg/g)	9.51 (5.08, 35.67)	12.88 (5.42, 39.69)	15.43 (7.20, 65.29)	0.002
Insulin therapy, n (%)	119 (38.26)	105 (33.76)	109 (35.04)	0.483
SGLT-2 inhibitors use, n (%)	16 (5.14)	18 (5.79)	18 (5.79)	0.922
Lipid lowering agents				
User of Fibrate, n (%)	10 (3.22)	12 (3.86)	13 (4.18)	0.812
User of Statin, n (%)	63 (20.25)	66 (21.22)	73 (23.47)	0.607
DKD prevalence, n (%)	92 (29.58)	100 (32.15)	116 (37.30)	0.114

ACEI, angiotensin converting enzyme inhibitor; ARB, angiotensin receptor blocker; DBP, diastolic blood pressure; SBP, systolic blood pressure; BMI, body mass index; HbA1c, glycated hemoglobin; LDL-C, low-density lipoprotein cholesterol; HDL-C, high-density lipoprotein cholesterol; TG, triglycerides; FBG, fasting blood glucose; TyG, triglyceride glucose; TyG-BMI, triglyceride glucose index × body mass index; eGFR, estimated glomerular filtration rate; ACR, albumin-to-creatinine ratio; DKD, diabetic kidney disease; SGLT-2, sodium-dependent glucose transporters 2.

### 3.2 Multivariate analyses of clinical factors associated with DKD

**Figure 1:**
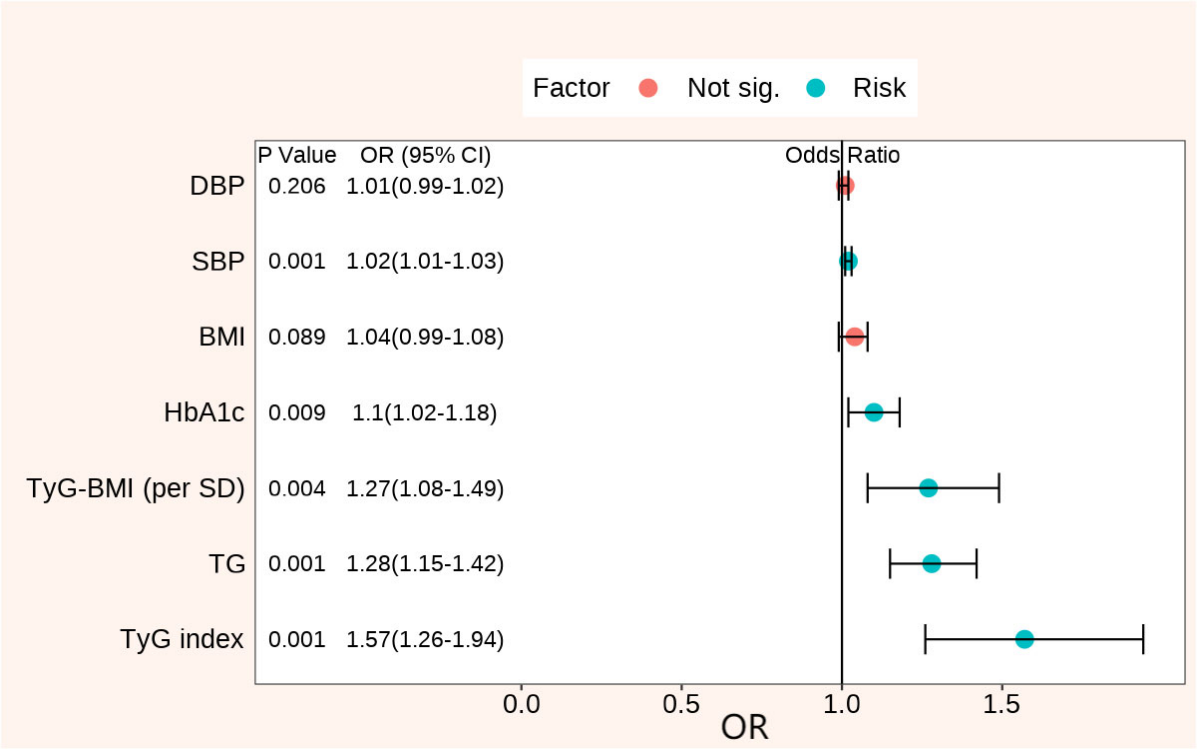
Forest plots of clinical factors associated with DKD adjusted for age, gender, hypertension, diabetic duration, and ACEI/ARB usage. DKD, diabetic kidney disease; DBP, diastolic blood pressure; SBP, systolic blood pressure; BMI, body mass index; HbA1c, glycated hemoglobin; ACEI, angiotensin converting enzyme inhibitor; ARB, angiotensin receptor blocker; TG, triglycerides; TyG, triglyceride glucose; TyG-BMI, triglyceride glucose index × body mass index; OR, odds ratio.

After adjusting for confounding factors such as age and gender, the multiple regression analysis revealed that SBP, DBP, BMI, HbA1c, TG, TyG index, and TyG-BMI were correlated with the occurrence of DKD in patients with T2D. SBP (OR, 1.02; 95% CI, 1.02–1.03; P < 0.001), HbA1c (OR, 1.10; 95% CI, 1.02–1.18; P= 0.009), TG (OR, 1.28; 95% CI, 1.15–1.42; P < 0.001), TyG index (OR, 1.57; 95% CI, 1.26–1.94; P < 0.001), and TyG-BMI (OR, 1.27; 95% CI, 1.08–1.49; P = 0.004) were still the independent risk factors for DKD after adjusting for age, gender, hypertension, diabetic duration, and ACEI/ARB usage (**Table 2**, **Figure 1**). Significantly, among the clinical factors, the TyG index exerted the greatest influence on the risk of DKD, each one-unit increase in the TyG index was associated with a 1.57-fold higher prevalence of DKD.

**Table 2:** Logistics analyses of clinical factors associated with DKD in patients with T2D.

Variables	Model I		Model II	
	OR (95%CI)	*P*	OR (95%CI)	*P*
SBP	1.02 (1.02- 1.03)	< 0.001	1.02 (1.01 - 1.03)	< 0.001
DBP	1.01 (1.00 - 1.03)	0.038	1.01 (0.99 - 1.02)	0.206
BMI	1.07 (1.03 - 1.11)	0.001	1.04 (0.99 - 1.08)	0.089
HbA1c	1.07 (1.00 - 1.14)	0.043	1.10 (1.02 - 1.18)	0.009
LDL-C	0.97 (0.82 - 1.14)	0.700	–	–
HDL-C	0.85 (0.54 - 1.34)	0.487	–	–
TG	1.27 (1.15 - 1.40)	< 0.001	1.28 (1.15 - 1.42)	< 0.001
FBG	1.04 (0.99 - 1.09)	0.136	–	–
TyG index	1.55 (1.26 - 1.90)	< 0.001	1.57 (1.26 - 1.94)	< 0.001
TyG-BMI (per SD)	1.37 (1.18 - 1.60)	< 0.001	1.27 (1.08 - 1.49)	0.004

Model I was adjusted for age and sex. Model II was adjusted for age, gender, hypertension, diabetic duration, and ACEI/ARB usage. ACEI, angiotensin converting enzyme inhibitor; ARB, angiotensin receptor blocker; DBP, diastolic blood pressure; SBP, systolic blood pressure; BMI, body mass index; HbA1c, glycated hemoglobin; LDL-C, low-density lipoprotein cholesterol; HDL-C, high-density lipoprotein cholesterol; FBG, fasting blood glucose; TG, triglycerides; TyG, triglyceride glucose; TyG-BMI, triglyceride glucose index × body mass index; DKD, diabetic kidney disease.

### 3.3 Univariate and multivariate analyses of DKD by TyG index tertiles

**Table 3** showed the association between the risk of DKD and the three tertiles of TyG index assessed by univariate and multivariate logistic regression analyses. After full adjustment (age, gender, hypertension, diabetic duration, ACEI/ARB usage, HbA1c, HDL-C, and LDL-C), compared to the lowest tertile of the TyG index, the increased risk of DKD in the tertile 2 group was not statistically significant [OR = 1.26 (0.85 - 1.85), P = 0.252]; however, individuals in the tertile 3 group were associated with a 91% higher prevalence of DKD [OR=1.91 (1.24 - 2.94), P = 0.003]. These results indicated that the risk of DKD did not increase linearly with rising TyG index levels.

**Table 3:** Univariate and multivariate logistic analyses of DKD in tri-sectional TyG index groups.

Variables	Non-adjusted		Adjust A		Adjust B	
	OR (95%CI)	*P*	OR (95%CI)	*P*	OR (95%CI)	*P*
Tertile 1	Ref.		Ref.		Ref.	
Tertile 2	1.13 (0.80 - 1.59)	0.488	1.28 (0.88 - 1.85)	0.193	1.26 (0.85 - 1.85)	0.252
Tertile 3	1.42 (1.01 - 1.98)	0.042	1.96 (1.34 - 2.87)	< 0.001	1.91 (1.24 - 2.94)	0.003
*P* for trend	0.115		0.002		0.01	

Adjust A was adjusted for age, gender, hypertension, diabetic duration, and ACEI/ARB usage; Adjust B was adjusted for age, gender, hypertension, diabetic duration, ACEI/ARB usage, HbA1c, HDL-C, and LDL-C. DKD, diabetic kidney disease; TyG, triglyceride glucose; LDL-C, low-density lipoprotein cholesterol; HDL-C, high-density lipoprotein cholesterol; HbA1c, glycated hemoglobin; ACEI, angiotensin converting enzyme inhibitor; ARB, angiotensin receptor blocker; OR, odds ratio.

### 3.4 TyG index non-linearly associated with DKD risk based on RCS analysis

**Figure 2:**
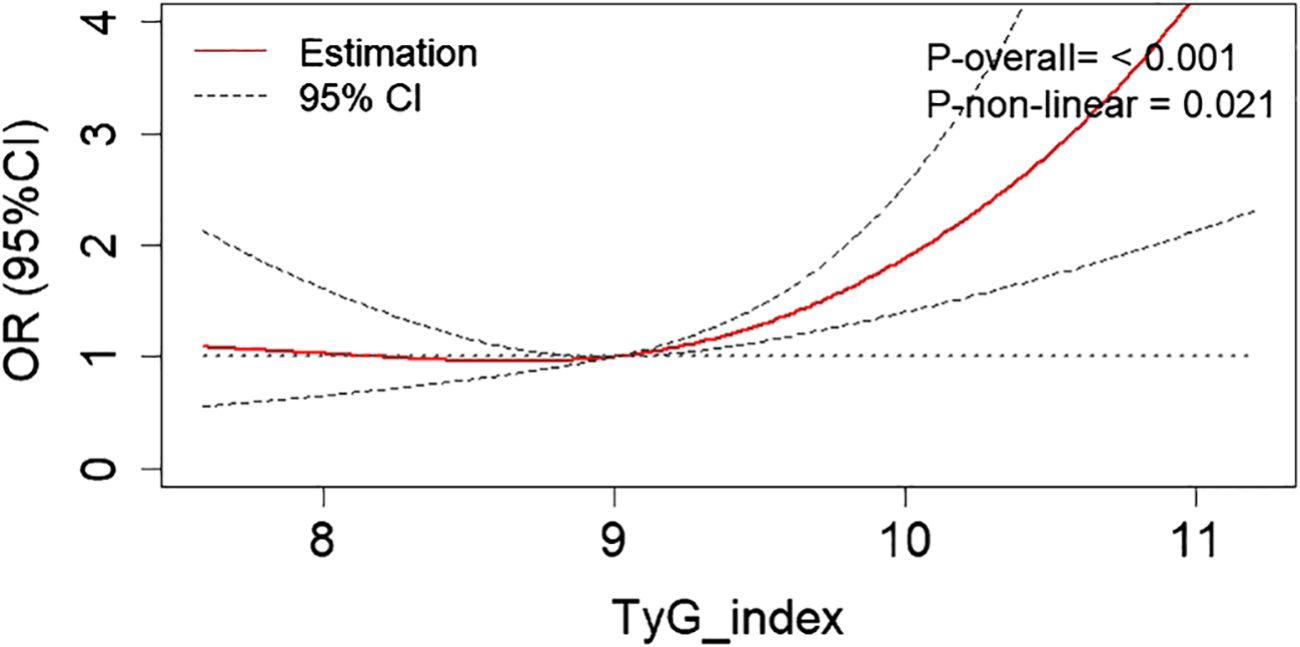
The RCS analysis revealed a non-linear relationship between the DKD risk and the TyG index after adjustment for age, gender, hypertension, diabetic duration, ACEI/ARB usage, HbA1c, HDL-C, and LDL-C. RCS, restricted cubic spline; DKD, diabetic kidney disease; TyG, triglyceride glucose; LDL-C, low-density lipoprotein cholesterol; HDL-C, high-density lipoprotein cholesterol; TG, triglycerides; HbA1c, glycated hemoglobin; ACEI, angiotensin converting enzyme inhibitor; ARB, angiotensin receptor blocker; OR, odds ratio.

RCS analysis revealed a non-linear relationship (P_non-linear_ = 0.021) between the risk of DKD and the TyG index after adjustment for age, gender, hypertension, diabetic duration, ACEI/ARB usage, HbA1c, HDL-C, and LDL-C (**Figure 2**). With the increase of the TyG index, the risk of DKD initially stabilized at a lower level, and then gradually rose. Based on the shape of this RCS curve, an optimal cut-off point of 9.35 for the non-linear relationship between TyG index and DKD risk was identified using the ‘CatPredi’ R package.

### 3.5 Threshold effect analyses of TyG index on the risk of DKD

The threshold effect analyses of TyG index on the risk of DKD in patients with T2D were summarized in **Table 4**. The participants with TyG index > 9.35 exhibited a 85% higher prevalence of DKD [OR= 1.85 (1.28 - 2.64), P < 0.001] compared to the subjects with TyG index ≦ 9.35. Analyzing the TyG index as a continuous variable revealed that among patients with TyG index ≦ 9.35, there was no statistically significant alteration in DKD risk with increasing TyG index [OR= 0.98 (0.61 - 1.58), P = 0.941]; however, for patients with a TyG index above 9.35, each one-unit increase in the TyG index was correlated with a 1.94-fold increase in the prevalence of DKD [OR= 1.94 (1.10 - 3.43), P < 0.022].

**Table 4:** Threshold effect analyses of TyG index on the risk of DKD in patients with T2D.

TyG index	Non-adjusted		Adjust A		Adjust B	
	OR (95%CI)	*P*	OR (95%CI)	*P*	OR (95%CI)	*P*
Categorical						
≦ 9.35	Ref.		Ref.		Ref.	
> 9.35	1.45 (1.08 - 1.94)	0.012	1.89 (1.36 - 2.63)	< 0.001	1.85 (1.28 - 2.64)	< 0.001
Continuous						
≦ 9.35	0.91 (0.62 - 1.34)	0.634	1.05 (0.69 - 1.61)	0.811	0.98 (0.61 - 1.58)	0.941
> 9.35	1.50 (0.94 - 2.39)	0.087	2.04 (1.19 - 3.50)	0.010	1.94 (1.10 - 3.43)	0.022

Adjust A was adjusted for age, gender, hypertension, diabetic duration, and ACEI/ARB usage; Adjust B was adjusted for age, gender, hypertension, diabetic duration, ACEI/ARB usage, HbA1c, HDL-C, and LDL-C. DKD, diabetic kidney disease; TyG, triglyceride glucose; LDL-C, low-density lipoprotein cholesterol; HDL-C, high-density lipoprotein cholesterol; HbA1c, glycated hemoglobin; ACEI, angiotensin converting enzyme inhibitor; ARB, angiotensin receptor blocker; OR, odds ratio; T2D, type 2 diabetes.

## 4 Discussion

The cross-sectional study confirmed a threshold effect between the TyG index and the risk of DKD. The results revealed that, after adjustment for confounding factors, the risk of DKD remained stable at a lower level among patients with a TyG index < 9.35, with no significant change as the TyG index increased; in contrast, for patients with a TyG index > 9.35, every one-unit rise in the TyG index led to a 94% increase in the risk of DKD. These findings could be valuable for the risk stratification and interventions in patients with T2D.

Insulin resistance has been shown to induce glucose metabolism disorders, oxidative stress, and inflammatory reactions, making it a significant contributor to several metabolic conditions, including diabetes and cardiovascular disease (22). It has been established as a predominant characteristic of T2D, with the identification of it holding significant clinical importance (22, 23). DKD is one of the most common complications caused by diabetes. Previous studies have demonstrated a strong association between the development of DKD and insulin resistance, potentially expediting the progression of DKD (24–26). Insulin resistance is related to multiple risk factors of DKD, such as dyslipidemia, central obesity, and hypertension; likewise, it may be exacerbated by the presence of DKD, indicating that impaired insulin sensitivity plays an important role in the pathogenesis and could be a potential treatment target of DKD (19). Hence, investigating the association between insulin resistance and DKD could lead to the development of targeted therapies aimed at reducing the risk of DKD, ultimately benefiting individuals with T2D and potentially enhancing their long-term outcomes. The homeostasis model is a commonly used indicator for assessing insulin resistance; however, it suffers from significant measurement variability due to the wide range of normal fasting serum insulin values and is further complicated by the effects of insulin therapy, rendering it impractical for both hospitalized and outpatient settings (19, 27). TyG index is a parameter calculated based on TG and FBG, used to assess insulin resistance (28). It is more economical and convenient compared to the gold standard method. The ability of the TyG index to evaluate insulin resistance is highly consistent with the gold standard method and is superior to the homeostasis model (17). Therefore, in this study, the TyG index was used as the indicator for evaluating insulin resistance.

In the present study, we observed a surprising discovery that individuals in the lowest tertile of the TyG index in T2D were older and had a longer duration of diabetes compared to those in the higher tertiles. This finding was consistent with a real-world study conducted by Wang S et al. (23). Currently, there is no other evidence to explain this phenomenon. Interestingly, after the patients were categorized into three groups based on their TyG index, no significant statistical differences were observed in the use of insulin and lipid-lowering drugs among these groups. This observation suggested that the impact of insulin and lipid-lowering medications on the overall TyG index trend within the type 2 diabetes population might have been relatively consistent or minor. Although these medications were effective in modifying lipid profiles, their direct impact on the TyG index—a marker derived from both triglyceride and glucose levels—appeared to be limited. This underscored the complex interplay between lipid metabolism and glucose homeostasis in diabetes management. This result aligned with findings from previous studies (23). However, further detailed investigations were needed to confirm or refine this observation, as it could have significant implications for the clinical management of diabetes.

Previous studies had shown that the TyG index was an independent risk factor for the risk of DKD (19, 29). In this study, similar results were obtained, with each one-unit increase in the TyG index being associated with a 1.57-fold higher prevalence of DKD after adjusting for confounding factors. Nonetheless, these findings simply suggested a general trend of increased risk of DKD with higher TyG index values, but did not provide insight into the dynamic relationship between them or confirm a linear association of DKD risk with escalating TyG index values. When the TyG index was used as a categorical variable in the logistic regression analysis, we found that the DKD risk associated with tertile 2 of the TyG index did not significantly differ from the DKD risk of tertile 1. This result was consistent with the findings of Mu X et al. (30), implying that DKD risk does not linearly increase with higher TyG index levels. Previous studies had used ROC analysis to determine the optimal cutoff values of the TyG index for diagnosing or predicting the occurrence of DKD, but the results indicated that the diagnostic performance of the TyG index was weak (AUC values respectively 0.62 and 0.57) (31, 32). Therefore, this study shifted its analytical focus to the value of the TyG index in stratifying the risk of DKD. Using the R package ‘CatPredi’, we calculated the optimal turning point for the nonlinear relationship between the TyG index and the risk of DKD, and conducted a threshold effect analysis. Ultimately, the risk of DKD stayed constant at a lower level for patients with a TyG index < 9.35; conversely, in patients with a TyG index > 9.35, each one-unit increase in the TyG index resulted in a 94% rise in the risk of DKD.

While our primary focus was on analyzing and highlighting the impact of the TyG index on DKD risk, it was crucial to recognize that the onset and progression of DKD were influenced by multiple factors. These included the duration of diabetes, glycemic control, and blood pressure levels, among other factors. The duration of diabetes was a well-known risk factor; longer duration of diabetes was associated with a higher risk of developing DKD (33). Similarly, the levels of glycemic and blood pressure control directly influenced the progression of microvascular damage (32). Assessing DKD risk from a comprehensive and broad perspective was essential for developing more effective strategies to manage and potentially mitigate DKD in patients with type 2 diabetes (34). Our findings suggested that while the TyG index served as a significant marker for insulin resistance and metabolic risk, the multifactorial nature of DKD necessitated a holistic approach to its management.

To the best of our knowledge, our study was the first investigation to reveal the threshold effect between the risk of DKD and the TyG index. Nonetheless, this study also has its limitations. One primary and significant limitation is the accuracy of the DKD definition used. In this study, DKD was defined based on a single measurement of albuminuria exceeding 30 mg/g or a eGFR below 60 ml/min/1.73 m^2^. However, according to clinical guidelines, a more reliable diagnosis usually requires at least two out of three consistent measurements during follow-up. Recognizing that both eGFR and albuminuria can be influenced by many factors not solely related to DKD, this cross-sectional design may limit the ability to accurately capture the chronicity and variability of these markers. Despite attempts to control for potential confounding factors, the possibility that unmeasured variables could affect the outcomes cannot be conclusively ruled out. Second, it is a cross-sectional study of single-center, the results may cause bias. Third, the detailed dosage information for the medications used by participants was absent, including insulin and lipid-lowering agents. The electronic medical records system utilized in our study only provided data on the history of medication use without specifying the dosages. This lack of detailed dosage information restricted our ability to fully assess the influence of these medications on the TyG index and their role in the management of type 2 diabetes. Finally, the findings can suggest an association between the TyG index and the prevalence of DKD in patients with T2D, but cannot assert predictive value. Future large-scale, multi-center prospective studies are needed to confirm the causality between TyG index and the risk of DKD in patients with T2D.

## 5 Conclusion

We found that there was a threshold effect between the prevalence of DKD and the increase of TyG index among the patients with T2D in China, which could be valuable for risk stratification and interventions in patients with T2D. Additionally, the TyG index, as an emerging marker that reflects insulin resistance, is simple, cost-effective, and reliable, showing significant promise for extensive application in primary care settings and communities. It can act as a valuable complement to the classic risk factors for DKD, offering further understanding of disease advancement and treatment approaches.

## Data availability statement

The raw data supporting the conclusions of this article will be made available by the authors, without undue reservation.

## Ethics statement

The studies involving humans were approved by Ethics Committee of the Affiliated Jinhua Hospital, Zhejiang University School of Medicine. The studies were conducted in accordance with the local legislation and institutional requirements. The ethics committee/institutional review board waived the requirement of written informed consent for participation from the participants or the participants’ legal guardians/next of kin because According to the regulations of the Ethics Committee, the consent for participation is not necessary for this retrospective cross-sectional study.

## Author contributions

HW: Conceptualization, Data curation, Formal analysis, Writing – original draft. GC: Data curation, Formal analysis, Writing – original draft. DS: Conceptualization, Data curation, Writing – original draft. YM: Conceptualization, Writing – review & editing.
